# Associations of smoking with cardiometabolic profile and renal function in a Romanian population-based sample from the PREDATORR cross-sectional study

**DOI:** 10.1080/13814788.2017.1324844

**Published:** 2017-06-09

**Authors:** Simona Georgiana Popa, Maria Moţa, Florin Dumitru Mihălţan, Adina Popa, Ioana Munteanu, Eugen Moţa, Cristian Serafinceanu, Cristian Guja, Nicolae Hâncu, Doina Catrinoiu, Radu Lichiardopol, Cornelia Bala, Bogdan Mihai, Gabriela Radulian, Gabriela Roman, Romulus Timar

**Affiliations:** ^a^ Department of Diabetes, Nutrition and Metabolic Diseases, University of Medicine and Pharmacy of CraiovaCraiovaRomania; ^b^ Pneumoftiziology Institute ‘Marius Nasta’ BucharestBucharestRomania; ^c^ Department of Diabetes, Nutrition and Metabolic Diseases, Clinical Emergency Hospital of CraiovaCraiovaRomania; ^d^ Department of Nephrology, University of Medicine and Pharmacy of CraiovaCraiovaRomania; ^e^ Department of Diabetes, University of Medicine and Pharmacy ‘Carol Davila’ BucharestBucharestRomania; ^f^ Department of Diabetes, University of Medicine and Pharmacy ‘Iuliu Haţieganu’ Cluj-NapocaCluj-NapocaRomania; ^g^ Faculty of Medicine, ’Ovidius’ University ConstanţaConstanţaRomania; ^h^ Department of Diabetes, University of Medicine and Pharmacy ‘Gr. T. Popa’ IaşiIaşiRomania; ^i^ Department of Diabetes, University of Medicine and Pharmacy ‘Victor Babeş’ TimişoaraTimişoaraRomania

**Keywords:** Epidemiology, smoking, cardiometabolic risk factors, PREDATORR study

## Abstract

**Background:** The impact of smoking on morbidity is well known, but in Romania, limited data are available regarding the smoking prevalence and relationship with cardiometabolic profile and kidney function.

**Objectives:** To assess the association of smoking with cardiometabolic traits and kidney function, in a Romanian population-based sample from the PREDATORR study.

**Methods:** PREDATORR was an epidemiological cross-sectional study. Between 2012 and 2014, participants were randomly selected from the lists of general practitioners and enrolled if they were aged 20 to 79 years, born and living in the past 10 years in Romania. Sociodemographic and lifestyle characteristics were collected through interviewer-administered questionnaires.

**Results:** Overall, 2704 participants were included in the analysis, 18% of them being current smokers and 30.8% former smokers. Current smokers compared to non-smokers had higher total cholesterol (220.6 ± 50.4 versus 213.9 ± 86.8 mg/dl, *P* = 0.017), LDL-cholesterol (137.8 ± 45.2 versus 130.7 ± 83.7 mg/dl, *P* = 0.004) and glomerular filtration rate (96.9 ± 16.8 versus 90.7 ± 19.1 ml/min/1.73 m^2^, *P* <0.001) in women and higher triglycerides (170.7 ± 129.8 versus 144.3 ± 94.2 mg/dl, *P* = 0.007), glomerular filtration rate (97.6 ± 17 versus 90.3 ± 18 ml/min/1.73 m^2^, *P* < 0.001) and lower HDL-cholesterol (48 ± 15.5 versus 50.4 ± 14.1 mg/dl, *P* = 0.002) in men. Active smoking was associated with hypercholesterolaemia [OR: 1.40 (95% CI: 1.01–1.96), *P* = 0.04] and low HDL-cholesterolaemia [OR: 1.39 (95% CI: 1.01–1.91), *P* = 0.04] and negatively associated with overweight/obesity [OR: 0.67 (95% CI: 0.48–0.94), *P* = 0.02]. Male former smokers had higher prevalence of abdominal obesity (82.4% versus 76.4%, *P* = 0.02), hypertriglyceridaemia (43.6% versus 35.6%, *P* = 0.01), hypertension (64% versus 56.4%, *P* = 0.01) and ischaemic vascular disease (40.5% versus 30.9%, *P* = 0.003) than male non-smokers.

**Conclusion:** The PREDATORR study showed a high prevalence of smoking in the adult Romanian population providing data on the association of smoking with cardiometabolic traits.

KEY MESSAGES

- PREDATORR was the first study in the Romanian population assessing smoking prevalence and its association with cardiometabolic disease and renal function.
- PREDATORR study showed a high prevalence of smoking in the adult Romanian population.
- PREDATORR study indicated an association of current smoking with unhealthy lifestyle and unfavourable lipid profile.

## Introduction

Smoking is a major risk factor for cardiometabolic diseases and chronic kidney disease (CKD). In 2000, it was estimated that smoking alone had caused over 650 000 deaths and serious chronic disease in 13 million people of the European Union [1]. Smoking is associated with the promotion and progression of atherosclerotic cardiovascular diseases and CKD [2–4].

Despite the known impact of smoking on morbidity and mortality, the prevalence of smoking is still high in Europe. In 2014, according to the Eurobarometer survey, the highest smoking prevalence in Europe was reported for Greece (38%) and the lowest for Sweden (11%), while in Romania the reported smoking prevalence was 27% [5].

A cross-sectional study conducted on both sides of the Hungarian-Romanian border reported a smoking prevalence of 36.4% in Romanian adults [6]. Romania is currently at stage 3 of the tobacco epidemic [7], characterized by a decline in the prevalence of male smokers and an initial decline followed by a plateau in female smokers [8].

In Romania, limited data are available regarding the smoking prevalence and association with cardiometabolic diseases and CKD. PREDATORR (prevalence of diabetes mellitus, prediabetes, overweight, obesity, dyslipidaemia, hyperuricaemia and chronic kidney disease in Romania) study indicated a high age and sex adjusted prevalence of diabetes (11.6%), obesity/overweight (31.9%/34.7%) and CKD (6.74%) in the Romanian adult population [9–11].

To our knowledge, PREDATORR is the first national study investigating the association of smoking with the cardiometabolic traits and kidney function in a sample representative for the adult Romanian population.

The present study is a secondary analysis of the PREDATORR study aiming to assess the association of smoking with cardiometabolic traits and kidney function, in a Romanian population-based sample.

## Methods

### Study design and participants

PREDATORR (EudraCT number: 2012-004803-12) was a national epidemiological cross-sectional study conducted from 2012 to 2014.

The PREDATORR survey was coordinated by the Romanian Society of Diabetes, Nutrition and Metabolic Diseases and the Romanian Society of Nephrology and was conducted according to the International Conference on Harmonisation—Good Clinical Practice Guidelines standards and World Medical Association Declaration of Helsinki—Ethical Principles for Medical Research Involving Human Participants (Seoul, 2008).

The study design was described elsewhere [9]. First, 101 general practitioners (GPs) were selected using automated random computer selection from the public database of the National Health Insurance Agency. Second, from the lists of the GPs, the study participants were randomly assigned to screening via a random number generator. The inclusion criteria were age between 20 and 79 years, born in Romania, living for the past 10 years mainly in Romania, included on the lists of a GP, no pregnancy/lactation. Overall, 2728 participants were enrolled based on the 2002 Romanian Census to have representativeness of the sample for the adult Romanian population. All participants provided written informed consent before any study procedure. The PREDATORR study was approved by the Romanian National Ethics Committee (Approval letter 4064/12.12.2012).

### Socio-demographic and lifestyle variables

Information about sociodemographic and lifestyle characteristics was collected through an interviewer-administered questionnaire. The educational level was categorized as medium/high (college, high school, university) or low (primary/secondary school). Participants were considered sedentary if they underwent physical activity less than four days/week. Participants who did not report consumption of alcoholic beverages during the last month were considered non-drinkers. Therapy non-compliance was considered when the participants missed taking the medication for more than six days in the last month.

Regarding smoking, participants were asked if they had ever smoked in their life, whether they currently smoke, the age at which they started/quit smoking and the number of cigarettes/day). The participants were classified according to smoking status as non-smokers (participants who never smoked), current smokers (participants who had smoked for longer than a year and had not stopped smoking) and former smokers (participants who quit smoking for more than one year).

### Clinical and biochemical measurements

The physical examination included measurement of anthropometric parameters, systolic (SBP) and diastolic blood pressure (DBP) using standard procedures. Participants with a body mass index (BMI) ≥ 25 kg/m^2^ were considered overweight/obese. Abdominal obesity was defined as a waist circumference ≥94 cm in men and ≥80 cm in women. Hypertension was defined as SBP ≥140 mmHg and/or DBP ≥90 mmHg and/or taking antihypertensive treatment or a personal history of hypertension. Diagnosis of ischaemic vascular disease (IVD)—coronary heart disease, carotid artery disease, peripheral arterial disease—was based either on self-report (personal history of stroke, myocardial infarction, gangrene, angioplasty, arterial by-pass) or medical records (ECG, arterial Doppler ultrasound, arteriography, etc.) from GPs and clinical examination.

All samples were collected in fasting state. Biochemical assays were performed at the Synevo Romania SRL laboratories. Fasting plasma glucose (FPG), total cholesterol (TC), HDL-cholesterol (HDL-C), triglycerides, creatinine and urinary creatinine levels were determined using enzymatic methods. Albuminuria and glycated haemoglobin (HbA1c) were measured using the immunoturbidimetric method and serum insulin was assessed with a chemiluminescent immunoassay. The concentrations of LDL-cholesterol (LDL-C) were calculated using the Friedewald formula if triglycerides <400 mg/dl. Insulin resistance (HOMA-IR) and insulin secretion (HOMA%B) were estimated using the equations proposed by Matthews et al. [12].

Impaired glucose regulation (IGR)—diabetes/prediabetes—was defined according to the American Diabetes Association guidelines 2012, using FPG, 2 h plasma glucose during the oral glucose tolerance test, HbA1c or self-reported diagnosis [13].

Hypercholesterolaemia was considered when TC ≥200 mg/dl and/or taking statins and high LDL-C when LDL-C ≥ 100 mg/dl and/or statin therapy. Low HDL-C was considered when HDL-C < 40 mg/dl in men or <50 mg/dl in women or treatment for reduced HDL-C and hypertriglyceridaemia when triglycerides ≥150 mg/dl or treatment for hypertriglyceridaemia.

CKD was defined as estimated glomerular filtration rate (eGFR) < 60 ml/min/1.73 m^2^ (CKD-EPI equation) and/or urinary albumin to creatinine ratio ≥30mg/g, according to the Kidney Disease Improving Global Outcomes 2012 guidelines [14].

### Statistical analysis

In the PREDATORR study sample, size calculation was performed only for the primary objective (determination of diabetes mellitus prevalence). Non-parametric tests were used for comparisons between smoking status of continuous and categorical variables.

Multivariate analysis by multinomial logistic regressions was performed to assess the association of cardiometabolic and renal parameters (dependent variables) with smoking status (independent variable). Odds ratio (OR) with 95% confidence interval (CI) are provided, and the non-smoker status was considered as a reference category. All analyses were stratified by sex due to the differences in smoking patterns by gender. *P* values <0.05 (two-tailed) were considered significant. Analyses were performed using SPSS software v19.0 (IBM Corp, Armonk, NY).

## Results

### Characteristics of the study population

Of the 2728 participants enrolled in the PREDATORR study, 2704 were included in the analysis by smoking status. The remaining 24 participants who stopped smoking within one year or had missing data on smoking status were excluded. The current smoking status frequency was 18% and the former smoking status frequency was 30.8%. Among the male participants, 22.1% were current smokers and 43.4% were former smokers, while only 14.3% of the female participants were current smokers and 19.3% were former smokers (*P* < 0.001 between the sexes for former smokers and, respectively current smokers).

Compared with former female smokers, the female current smokers had a longer duration of smoking (27.4 ± 11.8 versus 18.7 ± 12 years, *P* < 0.001) and started to smoke at a younger age (21.9 ± 6.7 versus 22.9 ± 6.5 years, *P* = 0.04). For males, current smokers had a longer duration of smoking compared with former smokers (30.5 ± 14.2 versus 23.2 ± 13.4 years, *P* < 0.001) but had lower daily consumption of tobacco (13.8 ± 7.5 versus 16.8 ± 10.7 cigarettes/day, *P* < 0.001).

In former smokers, males had longer a duration of smoking cessation compared with females (15.5 ± 12.3 versus 11.8 ± 10.1 years, *P* < 0.001).

The sociodemographic, lifestyle and anthropometric characteristics for the analysed sample are presented in Table 1.

**Table 1.** Sociodemographic, lifestyle and anthropometric data by smoking status.

	Female			Male		
	Non-smokers	Former smokers	Current smokers	Non-smokers	Former smokers	Current smokers
Total population, *n* (%)	941 (66.5)	273 (19.3)	202 (14.3)	444 (34.5)	559 (43.4)	285 (22.1)
Age (years), mean (SD)	57.8 (13.9)	53.8 (12.6)*	49.5 (12.5)#&	57.2 (14.5)	57.6 (13.1)	50.1 (14)#&
Medium/high educational level, %	76.6	88.6*	89.6#	88.2	88.7	93.0#
Marital status, %		*(overall)	#(overall)		*(overall)	#&(overall)
• Married	66.3	67.4	64.4	85.1	84.1	77.0
• Single	5.5	9.2	11.9	7.5	4.1	14.1
• Divorced	4.8	11.0	12.4	3.6	5.0	6.0
• Widowed	23.4	12.5	11.4	3.8	6.8	2.8
Alcohol drinking (yes), %	29.7	48.5*	45.5#	74.3	79.5	79.9
Sedentariness, %	19.9	18.0	20.4	15.9	17.5	22.3#
Therapy non-compliance, %	22.6	19.9	28.6&	22.7	22.3	31.5#&
BMI (kg/m^2^), mean (SD)	28.9 (5.8)	28.8 (6.3)	27 (5.7)#&	28.3 (4.4)	29 (5)*	27.2 (4.6)#&
Overweight/obesity, %	73.4	71.1	55.9#&	77.3	81.4	68.1#&
Waist (cm), mean (SD)	95.2 (14.7)	94.2 (15.8)	89.8 (14.9)#&	101.5 (13.4)	104.2 (13.1)*	100.2 (13.6)&
Abdominal obesity, %	85.8	81.3	71.1#&	76.4	82.4*	68.3#&

* *P* < 0.05 for former smokers versus non-smokers;

# *P* < 0.05 for current smokers versus non-smokers;

& *P* < 0.05 for current smokers versus former smokers;

%: percentage of participants; SD: standard deviation; BMI: body mass index; waist: waist circumference.

### Smoking and cardiometabolic profile

Current smokers had a worse lipid profile characterized by hypercholesterolaemia (*P* = 0.018) and high LDL-C (*P* = 0.017) in women and high triglycerides levels (*P* = 0.007) and low HDL-C (*P* = 0.013) in men compared to non-smokers (Table 2).

**Table 2.** Clinical and biological characteristics by smoking status.

	Female			Male		
	Non-smokers	Former smokers	Current smokers	Non-smokers	Former smokers	Current smokers
FPG (mg/dl), mean (SD)	91.4 (29.1)	91.8 (28.5)	86 (26)#&	94.8 (30.9)	97 (35.9)	89.5 (34.9)#&
HbA1c (%), mean (SD)	5.8 (2.6)	5.7 (0.8)	5.5 (0.8)#&	5.8 (0.9)	5.8 (1.1)	5.6 (0.9)#&
HOMA-IR, mean (SD)	4.5 (2.9)	2.7 (2.4)	2.6 (2.3)#&	4.7 (3)	3.1 (2.8)*	2.5 (2.4)#&
HOMA %B, mean (SD)	408.3 (254)	386 (238.5)	419.1 (301.6)	370.8 (242)	458.3 (252.8)	395.2 (244.1)
IGR, %	37.8	35.9	26.2#&	40.5	42.1	30.2#&
SBP (mmHg), mean (SD)	135.5 (23.2)	129.2 (20.9)*	129 (19.7)#	140.1 (19.3)	140.2 (19.6)	134.7 (18.6)#&
DBP (mmHg), mean (SD)	79.2 (12.2)	77.6 (11.7)	77.5 (11.2)	81.3 (11.5)	81.1 (12.6)	79 (11.8)#&
Hypertension, %	63.8	65.9	63.4	56.4	64.0*	63.7
TC (mg/dl), mean (SD)	213.9 (86.8)	214 (46.5)	220.6 (50.4)#	201.9 (43.5)	205.2 (48.2)	205.5 (45.3)
Hypercholesterolaemia, %	63.2	65.4	72.1#	60.0	64.3	62.8
TG (mg/dl), mean (SD)	125.5 (74)	129.4 (80.4)	132.2 (86)	144.3 (94.2)	166.6 (124.6)*	170.7 (129.8)#
Hypertriglyceridaemia, %	29.8	30.6	28.5	35.6	43.6*	41.4
HDL-C (mg/dl), mean (SD)	58.6 (18.3)	59.1 (15.2)	57 (16)	50.4 (14.1)	50 (15.1)	48 (15.5)#&
Low HDL-C, %	33.3	32.0	33.3	24.3	26.8	33.1#
LDL-C (mg/dl), mean (SD)	130.7 (83.7)	129.3 (41.1)	137.8 (45.2)#&	123.6 (37.6)	123.1 (40.6)	124.6 (39.3)
High LDL-C, %	76.9	80.9	84.8#	78.9	75.8	79.5
eGFR (ml/min/1.73 m^2^), mean (SD)	90.7 (19.1)	91.4 (19.7)	96.9 (16.8)#&	90.3 (18)	90.4 (18.2)	97.6 (17)#&
CKD, %	9.5	9.5	6.4	10.1	10.9	4.9 #&
IVD, %	30.5	29.0	20.8#	30.9	40.5*	26.7&

* *P* < 0.05 for former smokers versus non-smokers;

# *P* < 0.05 for current smokers versus non-smokers;

& *P* < 0.05 for current smokers versus former smokers;

%: percentage of participants; SD: standard deviation; FPG: fasting plasma glucose; HbA1c: glycated haemoglobin; HOMA-IR: insulin resistance; HOMA%B: insulin secretion; IGR: impaired glucose regulation; SBP: systolic blood pressure; DBP: diastolic blood pressure; HDL: high-density lipoprotein; LDL: low-density lipoprotein; eGFR: estimated glomerular filtration rate; CKD: chronic kidney disease; CKD-EPI: chronic kidney disease epidemiology; IVD: ischaemic vascular diseases.

Male former smokers had higher BMI (*P* = 0.04), and prevalence of abdominal obesity (*P* = 0.02), hypertriglyceridaemia (*P* = 0.013), hypertension (*P* = 0.016) and IVD (*P* = 0.03) than male non-smokers (Tables 1 and 2).

In both sexes, current smokers were younger and had lower anthropometric indices (waist circumference, BMI), better glucose regulation profile (FPG, HbA1c, HOMA-IR, the prevalence of IGR) and higher values of eGFR compared to non-smokers and former smokers (Tables 1 and 2).

#### Ischaemic vascular disease

Female current smokers compared to female non-smokers had a lower prevalence of IVD (*P* = 0.007) (Table 2). The prevalence of IVD was also lower in male current smokers than in former male smokers (*P* < 0.01) (Table 2).

#### Kidney function

Male current smokers had a lower prevalence of CKD compared with non-smokers (*P* = 0.012) and former smokers (*P* = 0.03) (Table 2).

The multinomial logistic regression, adjusted for covariates (sex, educational level, marital status, alcohol drinking, sedentariness), revealed that former smoker status was associated with the presence of IVD (*P* < 0.001) and hypertension (*P* = 0.026) (Table 3). Smoking cessation was negatively associated with age (*P* = 0.003).

**Table 3.** Factors associated with smoking status (multinomial logistic regression).

	Former smokers OR (95% CI)	Current smokers OR (95% CI)
Age	0.98 (0.97–0.99)**	0.96 (0.95–0.98)###
Overweight/obesity	0.97 (0.72–1.31)	0.67 (0.48–0.94)#
Abdominal obesity	1.05 (0.75–1.48)	0.97 (0.67–1.40)
IGR	1.01 (0.80–1.27)	0.99 (0.75–1.33)
Hypertension	1.26 (1.03–1.56)*	1.15 (0.89–1.47)
Hypercholesterolaemia	1.26 (0.95–1.69)	1.40 (1.01–1.96)#
Hypertriglyceridaemia	1.10 (0.84–1.44)	1.14 (0.82–1.59)
Low HDL-C	0.97 (0.74–1.26)	1.39 (1.01–1.91)#
High LDL-C	0.81 (0.58–1.12)	1.07 (0.72–1.58)
CKD	1.23 (0.87–1.74)	0.96 (0.59–1.55)
IVD	1.48 (1.18–1.86)***	1.07 (0.79–1.43)

The analysis was adjusted for covariates (sex, educational level, marital status, alcohol drinking, and sedentariness). ‘Non-smokers’ was considered the reference category.

* *P* < 0.05

** *P* < 0.01

*** *P* < 0.001 for former smokers versus non-smokers.

# *P* < 0.05

## *P* < 0.01

### *P* < 0.001 for current smokers versus non-smokers.

OR: odds ratio; CI: confidence interval; HDL: high-density lipoprotein; LDL: low-density lipoprotein; IGR: impaired glucose regulation; IVD: ischaemic vascular disease; CKD: chronic kidney disease.

Current smoker status was associated with hypercholesterolaemia (*P* = 0.04) and low HDL-C (*P* = 0.04) (Table 3). Current smoking was negatively associated with age (*P* < 0.001) and with the presence of overweight/obesity (*P* = 0.02) (Table 3).

## Discussion

### Main findings

According to our study, current smoking prevalence was 18% in the general Romanian population aged 20 to 79 years. An association of current smoking with hypercholesterolaemia and low HDL-C was identified in the present study, despite the fact that the current smokers were thinner and younger than non-smokers, suggesting a direct negative impact of smoking on the lipid profile. Our study indicated that compared to non-smokers current smokers had higher glomerular filtration rate in both sexes.

### Strengths and limitations

The main strengths of the present study are the representativeness of the sample from PREDATORR study for the Romanian population and the comprehensive diagnosis criteria of cardiometabolic diseases. Furthermore, we recorded important lifestyle characteristics, which improved our results due to the adjustment for these potential confounding factors that influence the cardiometabolic profile. Additionally, all laboratory measurements were performed within the same certified laboratory. Concerning the limitations, our study had a cross-sectional design; thereby we cannot investigate the mortality related to the smoking status, the future evolution of the cardiometabolic profile and kidney function and the causality relationship of smoking with the cardiometabolic traits and renal function.

### Prevalence of smoking

The current smoking prevalence found in PREDATORR study was lower than the prevalence reported by the SEPHAR study (27% in 2008) and Eurobarometer survey (27% in 2014) and Bunescu et al. [5,15,16], but these results should be interpreted taking into account the different structure of the population included in the studies.

### Smoking and cardiometabolic profile

Our results regarding smoking and cardiometabolic profile are consistent with previous findings that indicate that current smoking is involved in the pathological changes of all lipid fractions levels [2,17,18].

#### Body weight

The influence of smoking on body weight differs depending on smoking status. Several studies, as well as our study, showed that the BMI is lower in current smokers compared to non-smokers [19,20]. This could be explained by the younger age of current smokers as well as by the effects of nicotine, which increases energy expenditure and reduces appetite [19,21]. The results regarding BMI in former smokers (comparable to non-smokers in female and higher than non-smokers in male) may be explained by the variable duration of smoking cessation in female and male. Several studies indicated that smoking cessation is followed by weight gain in the first years after quitting; however, a few years later former smokers reach a weight that is comparable to never smokers [22]. Despite previous studies [23], current smokers in our study had a lower waist circumference compared to non-smokers probably due to the younger age of the current smokers compared to non-smokers, the advancing age being associated with a redistribution of fat tissue from subcutaneous to a visceral depot, without any change in the BMI [24]. Regarding the influence of smoking on blood pressure, several studies indicated that chronic smoking is associated with a lower blood pressure compared to non-smoking [18,25]. Primatesta et al. detected higher blood pressure values in male current smokers compared with non-smokers, but the independent chronic effect of smoking on blood pressure was small [26]. We found that smoking cessation was an independent predictor for hypertension, but we also detected a lower blood pressure in current smokers than in non-smokers and former smokers. An explanation for this may be the weight gain following smoking cessation, which could be accounted for the higher blood pressure observed in former smokers as well as younger age and lower weight of the current smokers [18].

#### Ischaemic vascular disease

In our study, the prevalence of IVD in female current smokers was lower than in female non-smokers. These conflicting results may be partially explained by the older age associated with a higher prevalence of overweight/obesity and IGR in female non-smokers, meaning that the female current smokers were less exposed to multiple IVD risk factors. These data are also emphasized by the results of the multinomial logistic regression (Table 3), which indicated that current smokers do not have less risk of IVD than non-smokers do. Furthermore, when analysing the discrepant findings regarding IVD between current and non-smokers we should consider the fact that only survivors are included in this cross-sectional study, thus, it may underestimate the strength of associations. Exposure to second-hand smoke was not included in the current study and this may also explain the conflicting findings, second-hand smoke being considered a health hazard [27]. The lower prevalence of IVD in male current smokers than in former male smokers revealed in our study may be explained, besides the younger age and better anthropometric and glycaemic profile by the lower daily consumption of tobacco in current smokers compared to former smokers.

#### Kidney function

The relationship between smoking and kidney function has not yet been well investigated [28,29]. Similar to another study, there was a higher eGFR in both male and female current smokers than in non-smokers and former smokers [29]. One plausible explanation is that nicotine induces intraglomerular hypertension followed by glomerular hyperfiltration [30].

### Implications for practice and research

The PREDATORR study, by showing an unexpectedly high prevalence of both current and former smoking in the adult Romanian population undergoing a rapid economic change, calls for an immediate action to introduce intervention procedures at population level. GPs have an important role in promoting the benefits of non-smokers status and in giving behavioural support to stop smoking.

PREDATORR study provides evidence that smoking is associated with cardiometabolic diseases and renal function impairment, therefore, in the current and former smokers, the cardiometabolic profile and renal function should be actively assessed to reduce the health and economic burden of these medical conditions.

## Conclusion

The PREDATORR study showed a high prevalence of smoking in the adult Romanian population providing data on the association of smoking with several cardiometabolic risk factors. Based on results of this study in Romanian participants, active smoking was associated with hypercholesterolaemia and low HDL-C despite the negative association with overweight/obesity while being a former smoker was associated with IVD and hypertension when compared with non-smokers.

Thereby smoking remains an important component of negative behaviour patterns, being a risk factor for cardiometabolic diseases.
